# Inductive Position and Speed Sensors

**DOI:** 10.3390/s20010065

**Published:** 2019-12-21

**Authors:** Pavel Ripka, Josef Blažek, Mehran Mirzaei, Pavol Lipovský, Miroslav Šmelko, Katarína Draganová

**Affiliations:** 1 Department of Measurement, Faculty of Electrical Engineering, Czech Technical University in Prague, Technicka 2, 16627 Prague 7, Czech Republic; mirzameh@fel.cvut.cz; 2 Faculty of Aeronautics, Technical University of Košice, Rampová 7, 041 21 Košice, Slovakia; josef.blazek@tuke.sk (J.B.); pavol.lipovsky@tuke.sk (P.L.); miroslav.smelko@tuke.sk (M.Š.); katarina.draganova@tuke.sk (K.D.)

**Keywords:** magnetic position sensors, magnetic speed sensors, magnetic trackers

## Abstract

Magnetic position and speed sensors are rugged and durable. While DC magnetic sensors use permanent magnets as a field source and usually have only mm or cm range, inductive sensors use electromagnetic induction and they may work up to a distance of 20 m. Eddy current inductive sensors equipped with magnetoresistive sensors instead of inductive coils can operate at low frequencies, allowing detection through a conductive wall. In this paper, we make an overview of existing systems and we present new results in eddy current velocity and position measurements. We also present several types of inductive position sensors developed in our laboratories for industrial applications in pneumatic and hydraulic cylinders, underground drilling, large mining machines, and for detecting ferromagnetic objects on conveyors. While the most precise inductive position sensors have a resolution of 10 nm and linearity of 0.2%, precision requirements on the industrial sensors which we develop are less demanding, but they should have large working distance and large resistance to environmental conditions and interference.

## 1. Introduction

Magnetic sensors of position, distance, and speed are reliable, precise, rugged, and durable. They are inexpensive and are, therefore, very popular for industrial, automotive, aerospace, security, and defense applications [1,2]. Unlike optical sensors, they are not sensitive to contamination, e.g., by grease or dust [3,4].

These sensors measure either linear or rotational motion and position. Their output may be bistable (this type of sensor is called a proximity switch), linear, encoded, or combined. Encoded output is either digital (absolute position sensors), pulse (incremental sensors), or pulse-width modulated (PWM). Combined outputs have a rough incremental scale and a fine linear scale [5].

The measured object (target) must be made of a ferromagnetic material for variable reluctance sensors and linear variable differential transformer (LVDT); the target should be conductive for eddy current sensors. The target can also bear a magnetic marker (either passive, such as LC resonator, or active, made by a transmission coil).

The two main types are DC magnetic position sensors with permanent magnet and induction sensors. DC magnetic position sensors are very popular for many industrial applications, requiring only mm or cm distance between the target and sensor. This paper is devoted to inductive position sensors which are based on magnetic induction law. Inductive sensors can work for distances from micrometers up to 20 m.

## 2. Eddy Current Inductive Sensors

An advantage of eddy current position and speed sensors is that they do not require a ferromagnetic target: Any conducting material can be used.

### 2.1. Eddy Current Position Sensors

Simple eddy current proximity sensors utilize the change in the AC coil impedance caused by the eddy currents in a conductive object in the vicinity of the coil. This coil is often a part of the LC oscillator, and the changes in impedance cause changes in the amplitude of the oscillations. The technologies behind eddy current (inductive) proximity sensors and linear displacement sensors are discussed in [6].

More precise eddy current position sensors consist of a transmission coil generating a primary AC magnetic field and a detecting coil, which senses the secondary magnetic field induced by the eddy current in the conducting target. In order to suppress the primary field, the detecting coil is perpendicular to the transmission coil, or two detecting coils are connected antiserially to measure the field difference.

In some cases, it makes sense to replace the detector coil with a DC magnetic sensor, e.g., an anisotropic magnetoresistor (AMR). Unlike in the case of induction coils, the sensitivity of AMR sensors is independent of frequency. This allows the use of low frequencies that can penetrate the metal wall of a pneumatic cylinder or barrel. Further advantages of AMR sensors are their small size and their low price, making them suitable for building sensor fields.

If the AC excitation and flipping frequencies are identical, the sensor has DC output without any detector [7]. When a DC magnetic sensor is used instead of an induction coil, the sensor is also able to distinguish ferromagnetic and non-magnetic targets. The main drawback of this type of sensor is that the output depends not only on the distance of the target, but also on its properties such as conductivity, permeability, and size. The dependence on the target thickness can be suppressed by using a high-enough excitation frequency, for which the penetration depth is significantly smaller than the minimum thickness of the target. In general, the eddy current distance sensors should be calibrated with each target.

High-performance sensors of this type use microprocessor or by Field-programmable gate array (FPGA) control electronics which performs non-linearity compensation and compensation of temperature. Table 1 gives the specifications of some of these devices.

### 2.2. Eddy Current Speed Sensors

An eddy current speed sensor based on a permanent magnet and a Hall sensor was designed for non-ferromagnetic moving objects without teeth or any other markers, e.g., a metal lining [8]. The sensitive axis of the Hall sensor is oriented orthogonally to the magnet field, so the sensor output is zero for zero speed. When the conducting body moves, eddy currents are generated, which is sensed by the Hall element. The sensor has several weak points that make it unsuitable for industrial applications: the stability of the primary field, the offset drift of the Hall sensor, its sensitivity to external magnetic fields, and its sensitivity to small angular mismatches caused by temperature dilatations.

**Figure 1:**
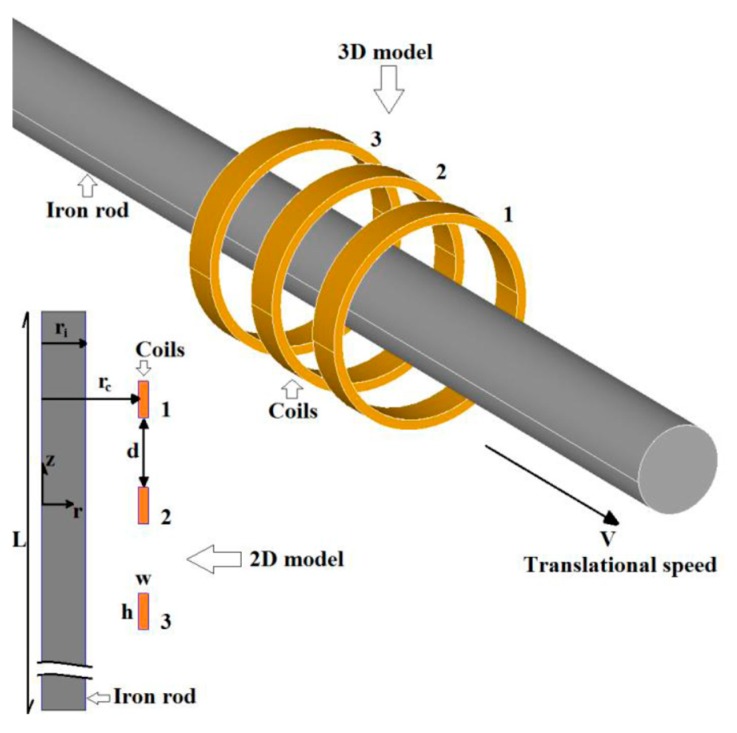
A coaxial eddy current linear speed sensor: sensor configuration with excitation coil in the middle and two sensing coils which are connected antiserially. For the developed demonstrator, w = 1.8 mm, h = 5 mm, d = 10 mm, and r = 10 mm. Reproduced from [9] with the permission of IEEE.

**Figure 2:**
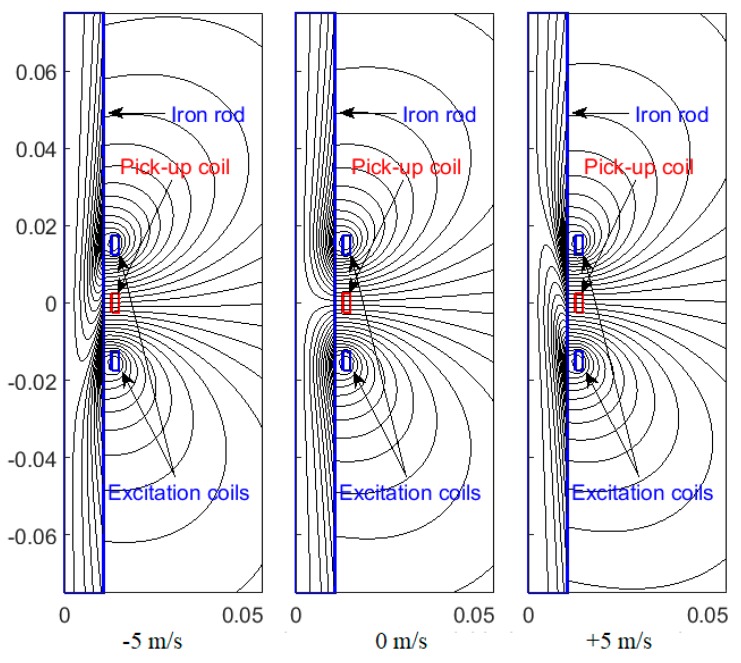
A coaxial eddy current linear speed sensor: Magnetic field distribution at zero speed and at a speed of ±5 m/s. The solid iron rod has relative permeability of 77 and conductivity of 4.45 MS/m.

**Figure 3:**
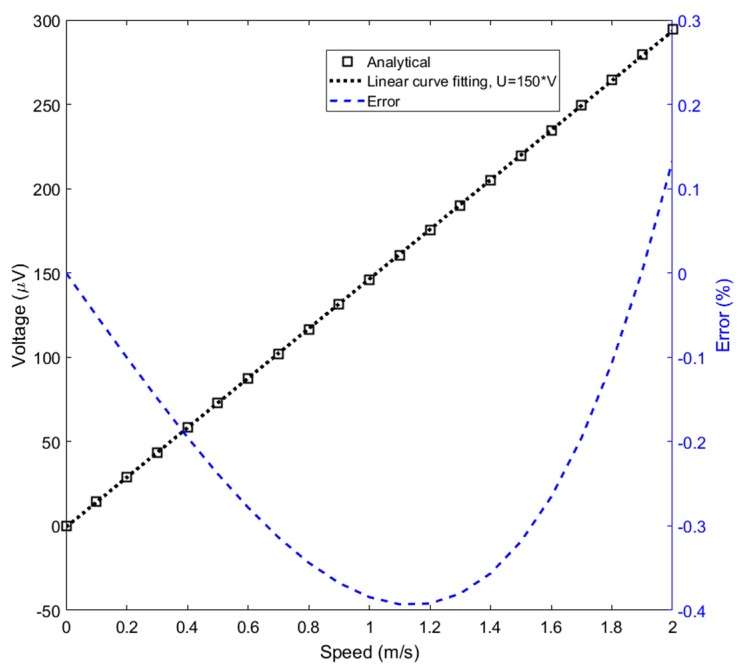
Characteristics and linearity error of the eddy current speed sensor. Analytical calculation assumes low magnetic field and thus constant initial permeability of the iron rod. Linearity relative error was calculated as %full-scale (FS).

A novel eddy current linear speed sensor has been developed for contactless measurements of the translational speed of an iron rod in linear machines and pneumatic and hydraulic cylinders [9]. Instead of a permanent magnet, the system uses an AC excitation coil and two antiserially connected pick-up coils (Figure 1). For zero speed the magnetic field is symmetrical, and thus the output voltage is zero. Due to the movement of the conducting core in the form of a rod, the eddy currents are distorted, and the resulting magnetic flux is no more symmetrical (Figure 2). Due to that, the output voltage appears, which is linearly proportional to the speed. The advantage of this sensor is its high linearity in a wide speed range. The sensor was tested for 20 mm diameter iron shaft and 47 turns of each coil; the achieved sensitivity was 150 μV/(m/s) and maximum linearity error was 0.4% (Figure 3).

A flat sensor of similar type uses rectangular excitation and sensing coils with axis perpendicular to the speed direction. The moving target is external to the coil. Such a setup may serve, for example, in the measurement of elevator or train speed using rails as moving part [10]. Typical sensitivity of 100 μV/(m/s) can be achieved with 100-turn coils. The sensitivity can be doubled by increasing the excitation frequency from 100 Hz to 1 kHz. However, increasing the operation frequency decreases the skin depth and increases the sensitivity to the surface properties of the moving part. Temperature compensation and compensation of the separation changes can be made by the ratiometric method using separate voltages of the output coils.

## 3. Linear Inductance and Transformer Sensors

A simple linear inductance position sensor uses a single solenoid with a movable core. The inductance of the coil depends on the position of the core. The linearity of this device can be improved by using varying winding density of the coil [11]. However, this simple sensor has large temperature dependence. The most popular sensor of this type is therefore the linear variable differential transformer, LVDT. It is based on a ferromagnetic core moving in a transformer with dual secondary windings, and it allows effective temperature compensation by ratiometric operation. Classical LVDT sensors have a coaxial shape, and, due to the rotational symmetry, they can be analyzed easily by 2D finite-element modeling (FEM).

An LVDT can use magnetic shielding for higher immunity against external magnetic fields [12]. Inductive position sensors can also be made ironless with moving coils—coaxial radiation-hard LVDT-like design is described in [13]. Instead of using the primary coil, it is possible to use only two coils together with inductance-to-voltage converters [14]. The most popular material for the LVDT sensor core is ferrite, but FeNi materials can also be used if mechanical robustness and low-temperature operation is required [15]. Differential transformer design allows simple temperature compensation, which provides 0.15% accuracy in a wide temperature range [16]. The temperature coefficient can be below 100 ppm/K. Devices with 0.1% linearity are available on the market. The linear range of LVDT can be significantly extended by digital techniques. Extension from 4 to 30 mm stroke was reported in [17] while linearity error was only 0.07%. A transformer position sensor was also used to measure the position of the piston in a pneumatic cylinder. Here the piston rod serves as an asymmetric moving core, and the coil system outside the piston and the low-frequency excitation field penetrates the conductive cylinder wall [18].

**Figure 4:**
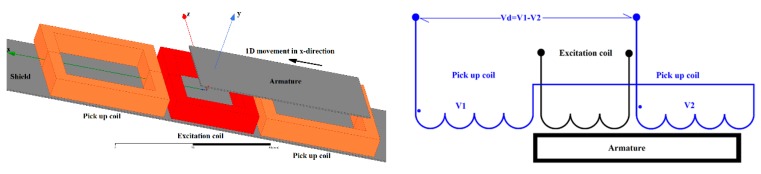
A flat linear transformer sensor consisting of stationary coil set with ferromagnetic shielding and moving armature.

**Figure 5:**
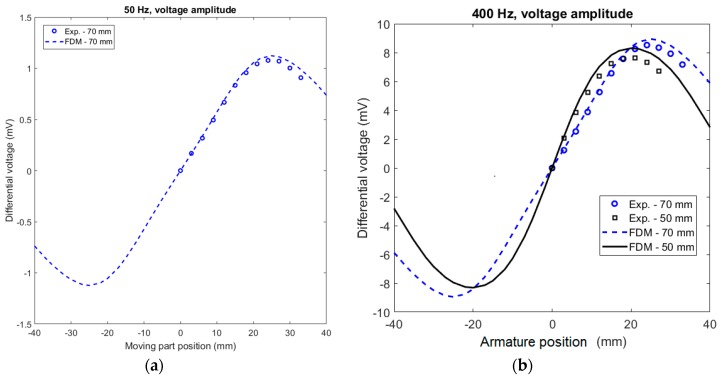
Differential voltage versus steel lamination armature position: (**a**) at f_exc_ = 50 Hz and (**b**) 400 Hz. Experimental results (Exp) versus calculations by finite-difference method (FDM). 2D FDM is a fast calculation method suitable for sensor optimization. The considered initial relative magnetic permeability, *µ*_r-I_ of steel lamination was 1000. Magnetic shielding was not considered.

While the basic configuration is coaxial with a moving rod core, flat devices are required for some applications. A planar design using flat coils appeared in [19,20], but these devices used a moving coil, which is very impractical for sensors with large strokes. We have designed a flat position sensor with a moving ferromagnetic armature and a stationary coil set, which consists of one excitation coil and two pick-up coils (Figure 4). The sensor can have a single moving target armature and multiple coil sets: a typical application is as a cabin landing sensor for elevators: The armature is connected to the cabin and one set of coils is located on each floor to signal the cabin landing position with sub-mm accuracy. With the use of a sensor 70 mm in length, 0.25 mm error without any compensation was achieved in a ±20 mm stroke (Figure 5).

**Figure 6:**
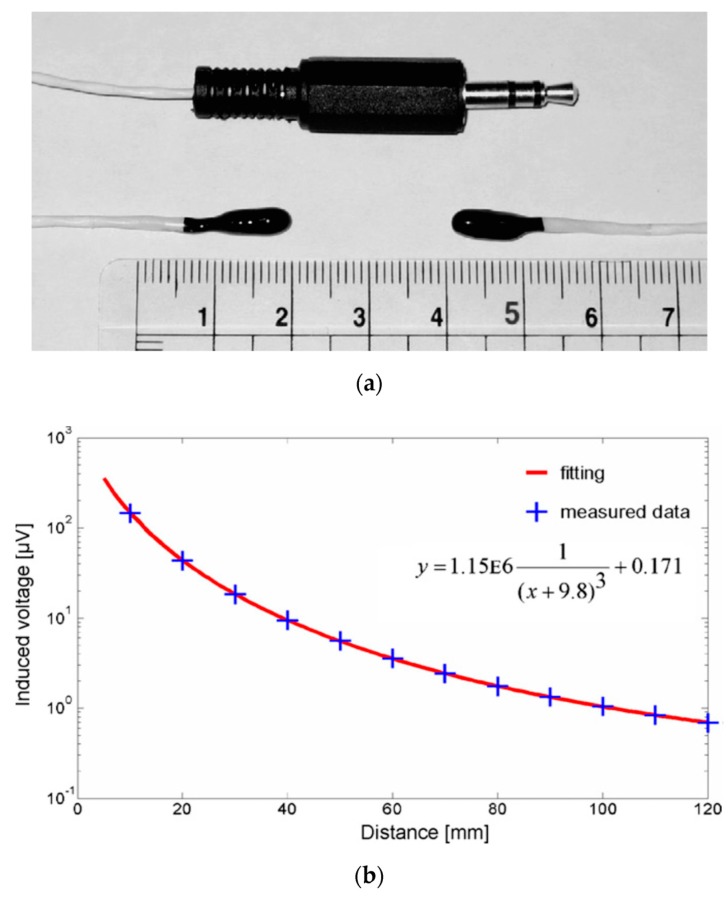
A system for measuring gastric activity in vivo. (**a**) A pair of implantable coils is attached to the gut; (**b**) output voltage vs. distance—from [22] with permission from Elsevier.

A position sensor based on the mutual inductance between two air coils is reported in [21]. The transmission coil is tuned and switched by a transistor. The position error is 1% for 240 mm distance. Similar sensors with solenoid coils developed for biomagnetic applications are described in [22]. A tri-axial miniature receiving coil was used to correct for angular mismatch between the transmitter and receiver coils. The system shown in Figure 6 was used to measure the gastric activity of dogs. The monitoring of the gut size and motion was previously performed using ultrasonic transmitters and receivers attached to the gut. Principal problem of using ultrasound inside the body is that the speed of sound is very different for different tissues and body liquids. Compared to that, magnetic properties of tissues are very similar. The sensitivity of the developed device was sufficient for detecting not only gut activity, but also breathing frequency. The achieved measurement range with probes placed coaxially was 10–120 mm with accuracy of 5% and resolution better than 1 mm. While such precision would be inacceptable for industrial applications, it is sufficient for medical research.

A precise distance sensor based on the principle of magnetic tracking is described in [23]. The system consists of a tri-axial stationary excitation coil system and a moving Billingsley tri-axial fluxgate magnetometer with 0.1 nT field resolution and linearity error below ±0.015% of the full scale. Each of three excitation coils is a 35 mm long, 22/35 mm in diameter, 300-turn solenoid. During one excitation cycle, the individual source coils are sequentially energized by current square wave pulses of both polarities to compensate for both the Earth’s field and sensor offset. The excitation frequency is reduced to 7 Hz in order to reduce the errors due to eddy currents in conducting structures in the vicinity of the measurement area. The achievable precision is 1% for any angular position between transmitter and receiver. With the use of a large excitation coil system and lower noise fluxgate sensors, the range can be extended up to 50 m.

## 4. Rotational Inductive Position and Speed Sensors

**Figure 7:**
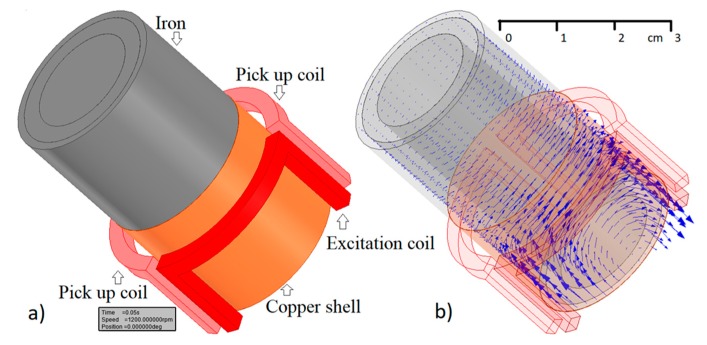
Rotational inductive speed sensor with copper shell, (**a**) geometry of an excitation and two pickup coils, (**b**) finite-element modeling (FEM) simulated magnetic flux at 1200 rpm speed. The solid iron shaft has a relative permeability of 100 and a conductivity of 5.54 MS/m. The 1 mm thick copper layer has a conductivity of 59.6 MS/m.

**Figure 8:**
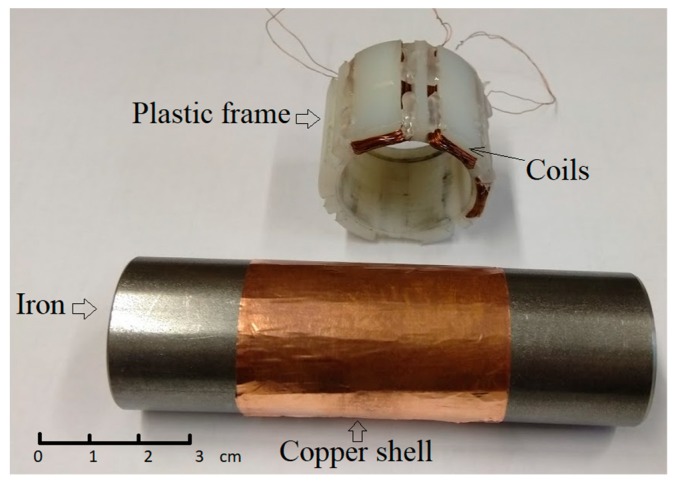
Rotational inductive speed sensor with copper shell.

Rotational inductive position and speed sensors are traditional devices based on stator and rotor coils. In some applications, e.g., in turbochargers, the use of a rotating coil is not practical or is even impossible. In this case, the eddy current principle can be utilized. A DC-excited system uses a stationary permanent magnet as a field source [8]. The limitation of this solution is again the temperature stability of the source field. An AC-excited rotational eddy current speed sensor consists of three stationary coils around the conductive shaft [24]. The sensor structure and magnetic field distribution are shown in Figure 7, while Figure 8 shows the prototype wound on a 3D printed bobbin. An advantage of this principle is its very high-speed range and high linearity due to the small working fields used. A disadvantage is that the sensitivity depends on the conductivity, which is temperature dependent. However, this effect can be easily corrected. Ferromagnetic shafts have low penetration depth and usually also low conductivity, resulting in low sensitivity at low speeds. This can be effectively compensated by using an aluminum or copper shell. Adding a 1 mm thick aluminum shell on top of a 28 mm diameter shaft would increase the sensitivity by a factor of 5: the resulting output is 100 μV/12,000 rpm at 90 Hz excitation and 50 turns of the sensing coil. The output voltage can be increased by increasing of the number of coil turns. For 1000 turns, the coil capacitance is still negligible at the used frequency and the increase of sensitivity would be by a factor of 400, as the output voltage is proportional to the multiple of the turn numbers.

## 5. Magnetometric Position Sensors and Magnetic Trackers

These devices use artificial AC or DC magnetic fields in a large volume to establish the position and the orientation of objects. The measured object often bears a 3D magnetometer, or sometimes only a resonant target, which is detected by a system of stationary coils or by magnetic sensors. Stationary magnetic markers are sometimes used to improve the precision. An artificial magnetic field usually has three perpendicular components, which are distinguished by different frequencies; some systems use time multiplexing to switch the direction of the artificial field. The applications of these systems include indoor, underground, and underwater navigation, and also virtual and mixed reality systems. Tracking systems are also used for surgery navigation [25,26].

A six-degree-of-freedom (DOF: three for position and three for orientation) wearable multisensory tracking system for virtual reality is described in [27]. This system is fusing data from an infrared optical tracker with a low-cost camera and an inertial and magnetic measurement unit. The hand posture can be tracked with 0.2 mm position error.

A five-DOF (without roll) indoor magnetic positioning system based on a single transmitting coil mounted on a moving object and three tri-axial magnetic sensors is described in [28]. The achieved accuracy is 10 cm and 6 deg.

## 6. Application Examples

In this section, we show several practical examples of systems developed for industrial and security applications. The magnetic sensing principle was selected for its robustness and often for the ability of the magnetic field to “see through the wall”.

### 6.1. Position Sensors for Pneumatic and Hydraulic Cylinders

A non-intrusive sensor for the position of a ferromagnetic piston in the cylinder of an aluminum combustion engine is described in [29]. The transducer utilizes the remnant magnetic field of the steel parts, which changes with time and temperature. This type of system therefore lacks long-term stability. A similar task is sensing the position of the piston inside a pneumatic cylinder. A challenge is posed by the shielding effect of the conducting aluminum cylinder. Direct mounting of the sensor inside the cylinder piston or piston rod is complicated and expensive [29,30]. An optical or magnetic scale on the piston rod is not reliable, and allows only incremental position sensing [31,32]. The use of a magnetoresistive delay line is not suitable, due to the harsh environment. The standard method is to use a permanent magnet attached to the piston and external magnetic sensors on the surface of the piston. This is a robust method, but it requires a non-magnetic stainless-steel piston rod and sensor array, which is expensive. A more suitable method uses a solenoid coil wound around the cylinder. The inductance of this coil depends on the position of the ferromagnetic piston and piston rod. A sensor of this type was developed only for hydraulic sensors with a non-conductive composite cylinder [33].

A transformer-based piston position sensor suitable for measurement through conducting cylinders is described in [18]. The linearity error is 1.6% without compensation. The traditional method of increasing linearity of the transformer position sensors at the coil ends is to modify the coil geometry by using uneven winding density or coil diameter. However, with using microprocessor or FPGA sensor controllers, software compensation using polynomial correction or look-up table is getting easier. The temperature dependence of this sensor is caused mainly by the change in the electrical resistivity of the aluminum cylinder, rather than by the permeability of the ferromagnetic parts. As the resistivity temperature dependence of aluminum is well known, it can be used for simple temperature compensation of the whole sensor.

### 6.2. Position Sensors for Underground Navigation

A tri-axial magnetometer is used as a magnetic compass for underground horizontal drilling. The azimuth is calculated using the pitch and roll angles measured by two inclinometers. This magnetometer cannot be replaced by other sensors such as gyros and accelerometers, as only sensor fusion can provide the required accuracy [34]. The Earth’s magnetic field is distorted by underground ferromagnetic objects, including drilling pipes. This error can be reduced by repeated measurements and multiple sensors [35].

Localization methods may use an artificial magnetic field. Liu uses a rotational permanent magnet on the surface and a tri-axial magnetometer at the drilling head [36]. The sensing range was limited to 5 m because of the AMR sensors that were used, but the position error was only 2%. The hybrid long-range tracking system for horizontal directional drilling uses an active magnetic ranger, an optical gyro, and three micromachined inclinometers [37]. The gyro is used for dead-reckoning navigation over kilometer-long distances. A magnetic tracking system consisting of two coaxial solenoid transmitters and two tri-axial magnetometer receivers is used when two drilling heads approach each other. By using only a longitudinal excitation field, the direction and distance can be calculated from two tri-axial readings. The magnetic approaching system has a maximum range of 17 m with 1.2 m rms accuracy, which is sufficient to steer the drilling heads. The accuracy increases by using larger number of the collected field data and also fast increases during the approach. At the distance of 10 m, the rms error is only 0.34 m, and the final approach is navigated with cm accuracy.

### 6.3. Position Sensors for Large Machines

**Figure 9:**
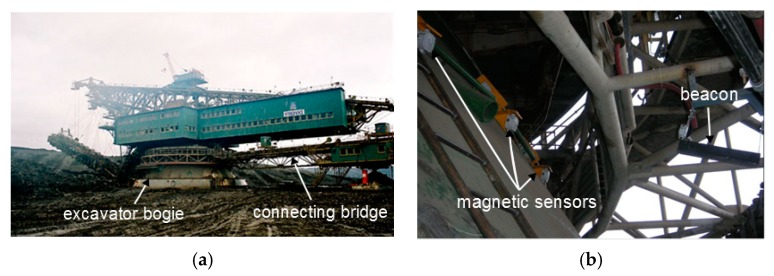
A rotational sensor for a walking crane, (**a**) the belly surrounded by the rotating structure, (**b**) a detail of the beacon and three of the 48 sensors.

An angular position sensor was required to measure the rotational angle between the excavator bogie and the connecting bridge of a walking crane. The rotating part is 33 m in diameter (Figure 9), and the environmental conditions are severe as regards temperature variations, vibrations, dust and mud, and electromagnetic interference. The active magnetic beacon is mounted on the moving part, whilst the stationary part is equipped with a field of 48 intelligent magnetic sensors [38]. The beacon source is a solenoid coil 60 cm in length and 5 cm in diameter with 800 turns, powered by a 2.5 A/50 Hz sinewave for 4 s. The minimum distance between the sensor and the beacon source is 1.5 m.

**Figure 10:**
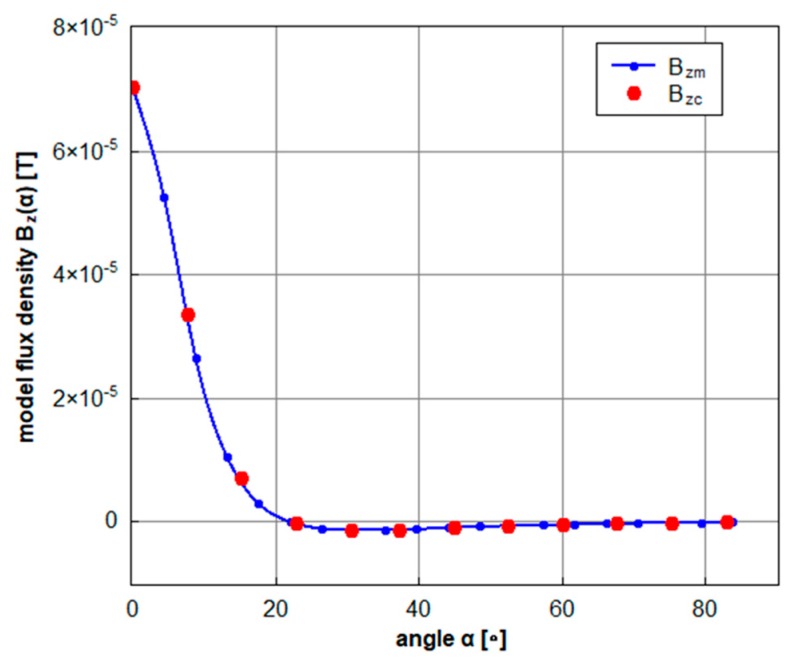
Magnetic field of the exciter for one quadrant of the angular position. B_zm_ is a field model calculated by FEM for a 2.5 A/50 Hz sinewave excitation and verified by the measurement. B_zc_ indicates the measured field values from sensors.

Figure 10 shows magnetic field of the exciter above the metal bell of the gear (as a function of the angular position of the sensors) for one quadrant of the measured angle. The exciter is placed in the 0° position above one of the sensors. The field values calculated by FEM and verified by the measurement form a model curve of B_zm_. The model is correlated with the measured values, B_zc_, from the sensors with the angular steps of 7.5° corresponding to the 48 sensors placed on the circumference. For the positions corresponding to the angle values higher than 75° the zero values were added to the model. The correlation is calculated 360 times with the model shift of one degree. The correct steer angle of the interconnecting bridge is than reliably determined by the sharp maximum of the correlation function.

### 6.4. Position and Velocity of Ferromagnetic Objects on Conveyors

Detection of moving ferromagnetic objects is required for protecting conveyor belts in surface coal mines. Other applications of this technology can be found in the food industry and in security applications such as detection and ranging of submersible vehicles. Speed measurements are required, e.g., in iron works and for monitoring road traffic.

**Figure 11:**
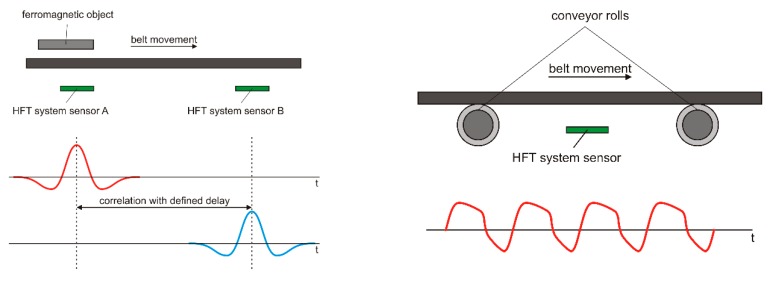
A moving ferromagnetic object and the stray field that is generated (**left**), and the stray fields from rollers (**right**).

**Figure 12:**
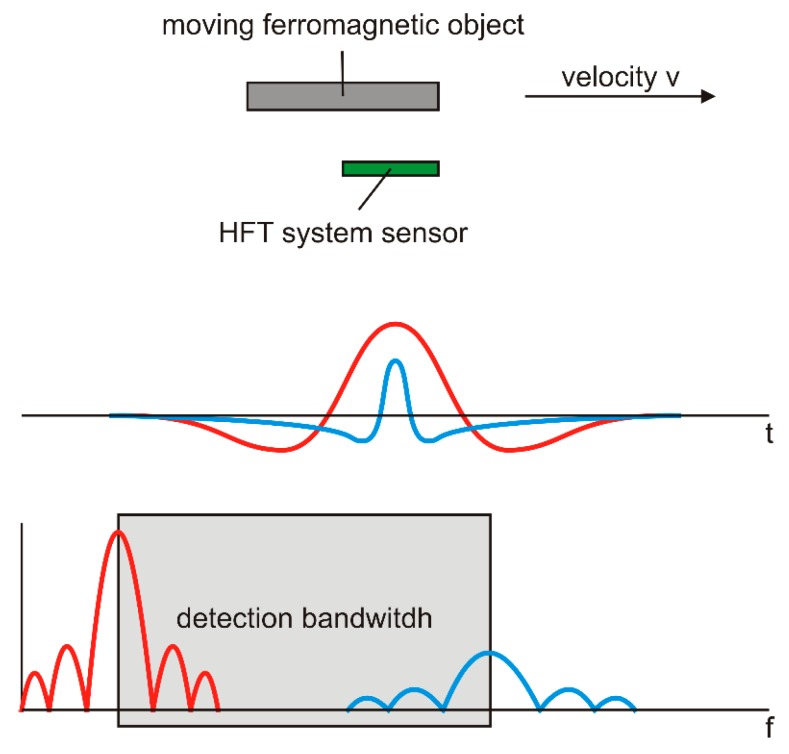
Cross-correlation between two channels of the system excludes random distant interferences.

Iron objects moving on the belt above the sensor generate a stray field which has a unique shape in the time and frequency domain depending mainly on the dimensions of the object [39]. Figure 11 shows a stray field of such an object. The bandwidth of the signal produced by iron objects 0.1–2 m in length, taking into consideration the speed of the belt 5 m/s, a distance of up to 1 m from the sensor, can be expected to be in the range from 1 to 20 Hz. In a typical industrial environment, this signal is almost hidden in the noise and in the interferences. The periodic interference in Figure 9 is produced by the moving ferromagnetic part of the conveyor structure—iron belt rolls and holders. These interferences are strong, because their sources are close to the sensors but their frequency is correlated to the speed of the belt. The interferences can therefore be almost fully removed by an adaptive comb filter. Random interference (noise) is caused by switching electric motors, lights, alarms, random vibrations of the structure, and by other moving objects. If the belt speed is known, the random interference can be suppressed by a correlation technique with the use of two or more sensors in a known distance (Figure 12). Alternatively, correlation analysis can be used to measure the speed of vehicles or moving ferromagnetic objects: The maximum of the correlation function corresponds to the best estimate of the transport delay.

### 6.5. Walking Detector

**Figure 13:**
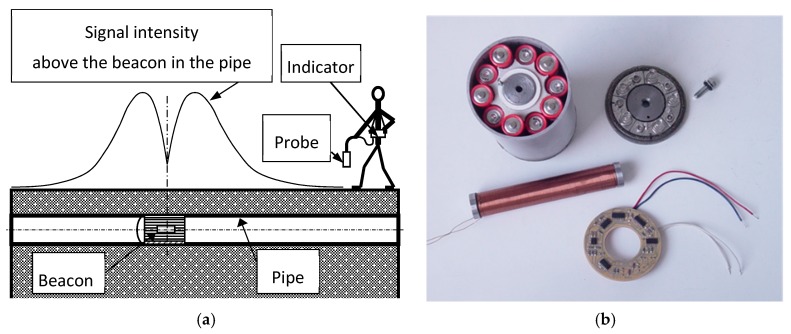
A walking detector for a magnetic pig, (**a**) schematics of the system, (**b**) beacon (transmitter) on the pig.

The walking detector is designed to find the position of a pig inside a pipe. The pig is equipped with a solenoid beacon (Figure 13) designed with the use of finite-element modeling (FEM). The solenoid has 3000 turns of a wire 0.35 mm in diameter with a core of soft iron 130 mm in length and 20 mm in diameter. The inductance of the solenoid is 0.94 H and its resistance is 25 Ω. The solenoid is energized by a 180 mA/12.5 Hz current.

**Figure 14:**
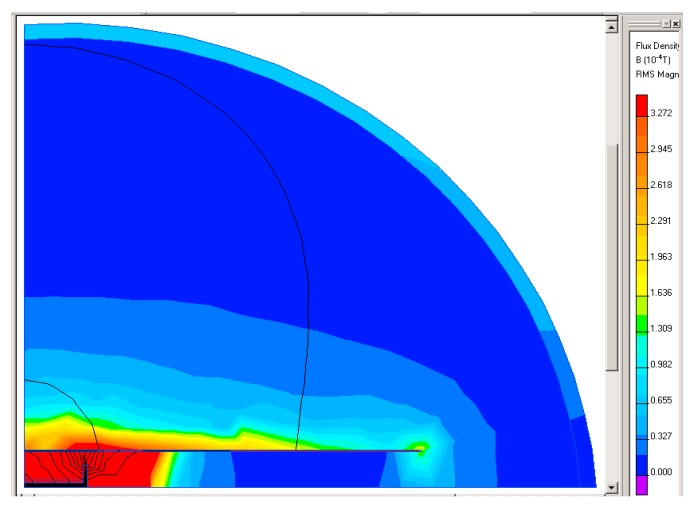
The field of the short solenoid inside the long ferromagnetic gas pipe calculated by FEM simulation. The considered relative permeability of the iron core was 1000.

**Figure 15:**
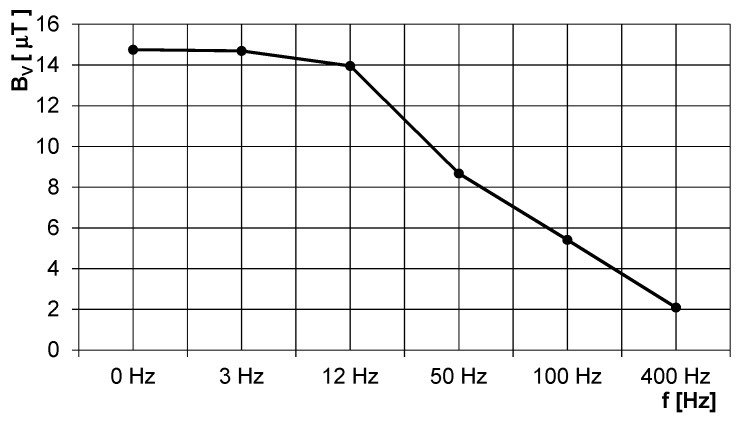
Magnetic flux density in 3 m distance from the pig inside the steel gas pipe as a function of the excitation field frequency: measured values.

**Figure 16:**
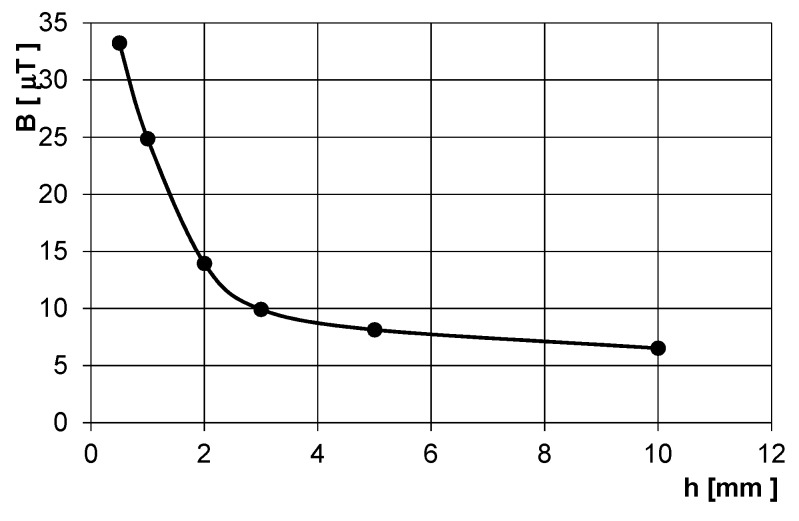
Magnetic flux density in 3 m distance from the pig inside the steel gas pipe as a function of the pipe wall thickness: measured values.

Figure 14 shows the field of the short solenoid inside the long ferromagnetic gas pipe with diameter of 100 mm and wall thickness of 2 mm. The working frequency is selected low in order to avoid field distortion due to eddy currents in the conductive parts and minimize the shielding effect of the steel pipe: Figure 15 shows steep decrease due to attenuation for frequencies above 12 Hz. Figure 16 shows attenuation at selected frequency of 12 Hz as a function of the pipe wall thickness: for thicknesses higher than 3 mm, this dependence is only mild. However, the excitation frequency is above the frequency of the walking person and, at the same time, far enough from 50 Hz.

**Figure 17:**
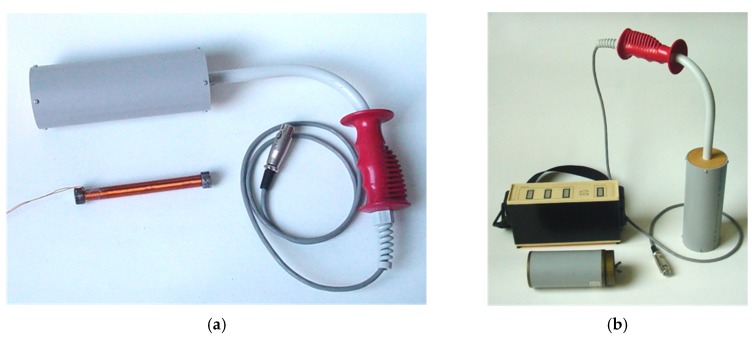
A walking detector sensor, (**a**) detail of the sensing coil and its core, (**b**) detector electronic box.

The beacon electronics is powered by 10 AA cells and works for 10 h from one set of batteries. The detection is performed by an induction coil with 200 turns working in the short-circuited (current) output (Figure 17). The detection limit can be selected between 1 and 100 nT, and for the pig located inside standard steel gas pipe, the detection distance is 5 m. In the case of plastic pipes, the pig signal is less attenuated and the detection distance is increased to 8 m. The positioning is accurate to ±0.2 m due to the sharp minimum of the signal.

## 7. Conclusions

Unlike magnetic encoders, which are based on DC field from permanent magnets and work only for small distances, inductive sensors use coil as an AC field source allowing large separation between the sensor and target. However, the most precise inductive eddy current may have 10 nm resolutions and 0.2% accuracy. The most popular inductive sensor of the transformer type is LVDT, which may achieve 150 nm repeatability and 0.15% accuracy. If the measurement is made for larger distances, the precision is limited: Angular sensor with 33 m diameter and 1.5 m separation described in Section 6.3 achieved only 1° accuracy, which is still sufficient for its application in walking crane. Magnetic navigation systems based on artificial fields may have 20 m range with cm precision.

Eddy current linear and rotational speed sensors can measure speed of conducting objects which have smooth surface. We achieved 0.4% linearity error for ferromagnetic target, while for non-ferrous target, the linearity is limited only by the electronics.

Performance of magnetic sensors is limited by Physics: A magnetic field in free space cannot be focused into a narrow beam and it penetrates all matter except superconductors. This limits the accuracy of magnetic sensors and their spatial resolution but gives them large resistance against dust and grease and also the ability to measure through walls.

## Figures and Tables

**Table 1:** Specification of the precise inductive distance sensors.

Type	Min Range (mm)	Max Range (mm)	Resolution (nm)	Drift (%FS/K)	Linearity (%FS)
Lion Precision U3	0.05	0.25	10	0.08	0.2
Lion Precision U50	2	15	300	0.02	0.2
µε eddyNCDT 3300 ES04	0.04	0.4	40	0.015	0.2
µε eddyNCDT 3300 EU80	8	80	4000	0.015	0.2
Kaman KD-5100 20N	0	1.9	100	0.025	10
Kaman KD-2306 60U1	0	60	6000	0.02	1

## References

[1] Zheng C., Zhu K., de Freitas S.C., Chang J.Y., Davies J.E., Eames P., Freitas P.P., Kazakova O., Kim C., Leung C.W. (2019). Magnetoresistive sensor development roadmap (non-recording applications). IEEE Trans. Magn..

[2] Reininger T., Welker F., von Zeppelin M. (2006). Sensors in position control applications for industrial automation. Sens. Actuators A Phys..

[3] Tumanski S. (2013). Modern magnetic field sensors—A review. Prz. Elektrotechniczny.

[4] George B., Tan Z.C., Nihtianov S. (2017). Advances in capacitive, eddy current, and magnetic displacement sensors and corresponding interfaces. IEEE Trans. Ind. Electron..

[5] Feng T., Hao S.H., Hao M.H., Wang J.L. (2016). Development of a combined magnetic encoder. Sens. Rev..

[6] Fericean S., Droxler R. (2007). New noncontacting inductive analog proximity and inductive linear displacement sensors for industrial automation. IEEE Sens. J..

[7] Ripka P., Vyhnanek J., Janosek M., Vcelak J. (2014). AMR proximity sensor with inherent demodulation. IEEE Sens. J..

[8] Cardelli E., Faba A., Tissi F. (2013). Contact-less speed probe based on eddy currents. IEEE Trans. Magn..

[9] Mirzaei M., Ripka P., Chirtsov A., Vyhnanek J. (2019). Eddy current linear speed sensor. IEEE Trans. Magn..

[10] Mirzaei M., Ripka P., Chirtsov A., Vyhnanek J., Grim V. (2020). Design and modeling of a linear speed sensor with a flat type structure and air coils. J. Magn. Magn. Mater..

[11] Yang S.H., Hirata K., Ota T., Kawase Y. (2017). Impedance linearity of contactless magnetic-type position sensor. IEEE Trans. Magn..

[12] Martino M., Danisi A., Losito R., Masi A., Spiezia G. (2010). Design of a linear variable differential transformer with high rejection to external interfering magnetic field. IEEE Trans. Magn..

[13] Grima A., Di Castro M., Masi A., Sammut N. (2018). Electrical metrological characterization of ironless inductive position sensors with long cables. IEEE Sens. J..

[14] Mandal H., Bera S.K., Saha S., Sadhu P.K., Bera S.C. (2018). Study of a modified lvdt type displacement transducer with unlimited range. IEEE Sens. J..

[15] Yanez-Valdez R., Alva-Gallegos R., Caballero-Ruiz A., Ruiz-Huerta L. (2012). Selection of soft magnetic core materials used on an lvdt prototype. J. Appl. Res. Technol..

[16] Petchmaneelumka W., Rerkratn A., Luangpol A., Riewruja V. (2018). Compensation of temperature effect for lvdt transducer. J. Circuits Syst. Comput..

[17] Petchmaneelumka W., Koodtalang W., Riewruja V. (2019). Simple technique for linear-range extension of linear variable differential transformer. IEEE Sens. J..

[18] Ripka P., Mirzaei M., Chirtsov A., Vyhnanek J. (2019). Transformer position sensor for a pneumatic cylinder. Sens. Actuators A Phys..

[19] Djuric S.M. (2014). Performance analysis of a planar displacement sensor with inductive spiral coils. IEEE Trans. Magn..

[20] Anandan N., George B. (2017). Design and development of a planar linear variable differential transformer for displacement sensing. IEEE Sens. J..

[21] Laskoski G.T., Pichorim S.F., Abatti P.J. (2012). Distance measurement with inductive coils. IEEE Sens. J..

[22] Tomek J., Mlejnek P., Janasek V., Ripka P., Kaspar P., Chen J. (2008). The precision of gastric motility and volume sensing by implanted magnetic sensors. Sens. Actuators A Phys..

[23] Zikmund A., Ripka P. (2012). A magnetic distance sensor with high precision. Sens. Actuators A Phys..

[24] Mirzaei M., Ripka P., Vyhnanek J., Chirtsov A., Grim V. (2019). Rotational eddy current speed sensor. IEEE Trans. Magn..

[25] Attivissimo F., Lanzolla A.M.L., Carlone S., Larizza P., Brunetti G. (2018). A novel electromagnetic tracking system for surgery navigation. Comput. Assist. Surg..

[26] Sha M., Wang Y.F., Ding N., Wu X.M., Fang Z.X. (2017). An electromagnetic tracking method based on fast determination of the maximum magnetic flux density vector represented by two azimuth angles. Measurement.

[27] Maereg A.T., Secco E.L., Agidew T.F., Reid D., Nagar A.K. (2017). A low-cost, wearable Opto-Inertial 6-DOF hand pose tracking system for VR. Technologies.

[28] Hehn M., Sippel E., Carlowitz C., Vossiek M. (2019). High-accuracy localization and calibration for 5-dof indoor magnetic positioning systems. IEEE Trans. Instrum. Meas..

[29] Taghvaeeyan S., Rajamani R., Sun Z.X. (2013). Non-Intrusive piston position measurement system using magnetic field measurements. IEEE Sens. J..

[30] Taghvaeeyan S., Rajamani R. (2015). Magnetic sensor-based large distance position estimation with disturbance compensation. IEEE Sens. J..

[31] Faudzi A.M., Suzumori K., Wakimoto S. Design and control of new intelligent pneumatic cylinder for intelligent chair tool application. Proceedings of the IEEE/ASME International Conference on Advanced Intelligent Mechatronics.

[32] Yang S.Y., Lee M.C., Lee M.H., Arimoto S. (1998). Measuring system for development of stroke-sensing cylinder for automatic excavator. IEEE Trans. Ind. Electron..

[33] Sumali H., Bystrom E.P., Krutz G.W. (2003). A displacement sensor for nonmetallic hydraulic cylinders. IEEE Sens. J..

[34] Wang Z.H., Poscente M., Filip D., Dimanchev M., Mintchev M.P. (2013). Rotary in-drilling alignment using an autonomous MEMS-based inertial measurement unit for measurement-while-drilling processes. IEEE Instrum. Meas. Mag..

[35] Park B., Myung H. (2018). Resilient underground localization using magnetic field anomalies for drilling environment. IEEE Trans. Ind. Electron..

[36] Liu T., Wang B.X. (2014). Guidance method in HDD based on rotating magnetic field. IEEE Trans. Geosci. Remote Sens..

[37] Vcelak J., Ripka P., Zikmund A. (2015). Long-range magnetic tracking system. IEEE Sens. J..

[38] Blazek J., Praslicka D., Hudak J., Klinda A., Mikita I., Marcin J. (2010). New generation of magnetic relaxation sensors based on the melt-spun FeCoBCu alloys. Acta Phys. Pol. A.

[39] Praslicka D., Blazek J., Hudak J., Mikita I., Moucha V. (2015). Industrial applications of magnetometry. J. Electr. Eng. Elektrotechnicky Cas..

